# Site-Directed Mutagenesis of the Fibronectin Domains in Insulin Receptor-Related Receptor

**DOI:** 10.3390/ijms18112461

**Published:** 2017-11-19

**Authors:** Igor E. Deyev, Natalia A. Chachina, Egor S. Zhevlenev, Alexander G. Petrenko

**Affiliations:** 1 Group of Molecular Physiology, Institute of Bioorganic Chemistry, Shemyakin—Ovchinnikov Institute of Bioorganic Chemistry Russian Academy of Sciences, 117997 Moscow, Russia; zhevlenev.egor@mail.ru; 2 Laboratory of Receptor Cell Biology, Institute of Bioorganic Chemistry, Shemyakin—Ovchinnikov Institute of Bioorganic Chemistry Russian Academy of Sciences, 117997 Moscow, Russia; n_chachina@inbox.ru (N.A.C.); petrenkoag@gmail.com (A.G.P.)

**Keywords:** receptor tyrosine kinase activation, tyrosine phosphorylation, protein glycosylation, site directed mutagenesis, insulin receptor-related receptor, alkaline medium, pH sensor

## Abstract

The orphan insulin receptor-related receptor (IRR), in contrast to its close homologs, the insulin receptor (IR) and insulin-like growth factor receptor (IGF-IR) can be activated by mildly alkaline extracellular medium. We have previously demonstrated that IRR activation is defined by its extracellular region, involves multiple domains, and shows positive cooperativity with two synergistic sites. By the analyses of point mutants and chimeras of IRR with IR in, we now address the role of the fibronectin type III (FnIII) repeats in the IRR pH-sensing. The first activation site includes the intrinsically disordered subdomain ID (646–716) within the FnIII-2 domain at the C-terminus of IRR alpha subunit together with closely located residues L135, G188, R244, H318, and K319 of L1 and C domains of the second subunit. The second site involves residue T582 of FnIII-1 domain at the top of IRR lambda-shape pyramid together with M406, V407, and D408 from L2 domain within the second subunit. A possible importance of the IRR carbohydrate moiety for its activation was also assessed. IRR is normally less glycosylated than IR and IGF-IR. Swapping both FnIII-2 and FnIII-3 IRR domains with those of IR shifted beta-subunit mass from 68 kDa for IRR to about 100 kDa due to increased glycosylation and abolished the IRR pH response. However, mutations of four asparagine residues, potential glycosylation sites in chimera IRR with swapped FnIII-2/3 domains of IR, decreased the chimera glycosylation and resulted in a partial restoration of IRR pH-sensing activity, suggesting that the extensive glycosylation of FnIII-2/3 provides steric hindrance for the alkali-induced rearrangement of the IRR ectodomain.

## 1. Introduction

Insulin receptor-related receptor (IRR) belongs to the insulin receptor minifamily of receptor tyrosine kinases that also includes the insulin receptor (IR) and insulin-like growth factor receptor (IGF-IR). [1,2,3,4]. All three receptors are highly homologous by their amino acid sequence and exhibit similar domain structure. Unlike other receptor tyrosine kinases that activate due to receptor dimerization upon ligand binding [5], these three receptors are expressed in the pre-dimerized form where subunits are already linked by disulfide bonds, and their activation involves a major conformational change that leads to transphosphorylation of their intracellular tyrosine kinase domains [6,7]. Due to endogenous proteolytic processing of the precursor protein, mature receptor monomers consist of the disulfide-linked hydrophilic extracellular α and membrane-spanning β-subunits, the latter bearing an intracellular catalytic phosphotyrosine kinase domain [8].

As of now, no peptide or protein has been yet found to activate IRR. Unexpectedly, an unusual property of IRR to work as a sensor of extracellular medium pH was discovered [9,10]. It was proposed that the hydroxyl ion is an agonist of IRR because IRR activation by alkaline media is specific with regards to IR and IGF-IR, dose-dependent and reversible. Also, IRR triggers the intracellular signaling and cytoskeleton remodeling [11,12,13]. In contrast with insulin binding to IR, the activation of IRR demonstrates positive cooperativity with Hill coefficient around 2.3 [14]. IRR knockout mice did not show any overt phenotype under normal conditions [15] whereas knockouts of IR and IGF-IR are lethal [16,17]. Yet, the triple knockout of all the members of the insulin receptor minifamily suggested the role of IRR in the reproductive system development by an unknown mechanism [16].

The pH-sensing property of IRR is preserved in frog, mouse, and human [10], while the amino acid sequences of the IR minifamily members are highly conserved in species from amphibians to humans [18]. Unlike its close homologs, IRR shows highly specific tissue distribution primarily in organs that are involved in acid/base production (stomach, kidney, and pancreas) [15,19]. In vivo experiments in knockout mice revealed the role of IRR in the regulation of bicarbonate excretion by the kidneys under experimentally-induced alkalosis [10,20].

By the analysis of IRR/IR chimeras and IRR point mutants, it was shown that the alkali-induced activation of IRR is defined by its extracellular region and involves multiple domains with the key role of L1C domains and FnIII repeats [14,21,22]. This study was focused on the role of conserved amino acid residues within fibronectin type-III repeats regions of IRR (FnIII-1, FnIII-2, and FnIII-3). We also addressed the question of how glycosylation in FnIII-2/3 may influence pH sensitivity of IRR.

## 2. Results

**Figure 1:**
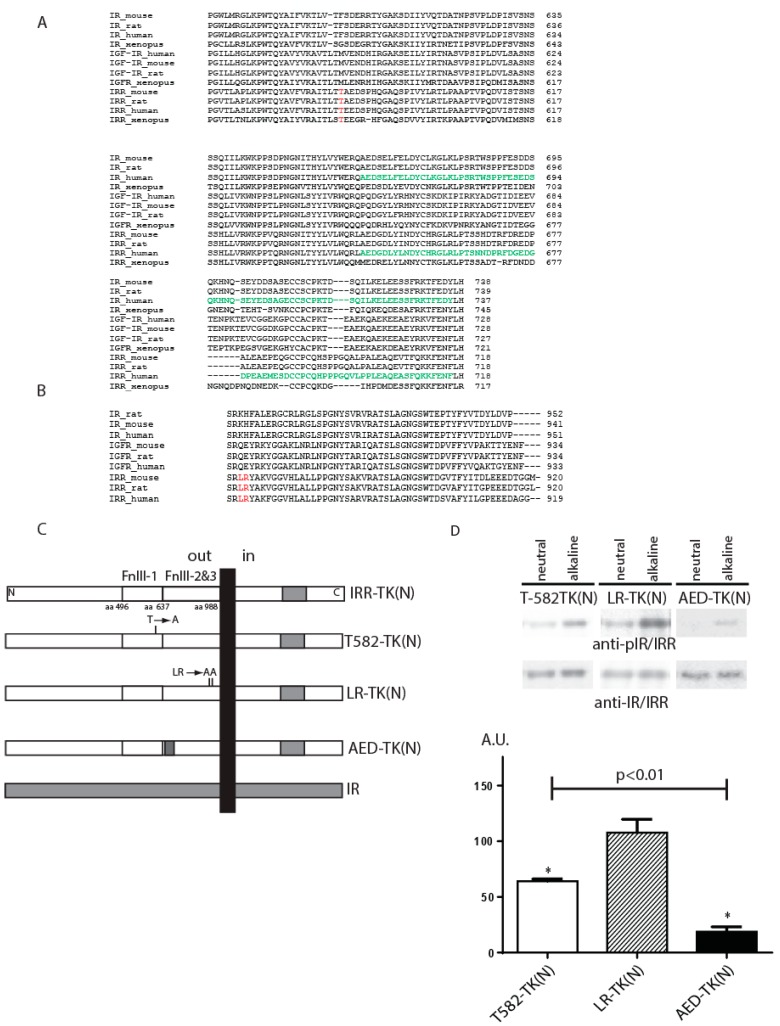
The role of T582, L868, R869 (red) and region (646–716) (green) within fibronectin repeats domains. (**A**) Alignment of insulin receptor (IR), insulin-like growth factor receptor (IGF-IR) and insulin receptor-related receptor (IRR) sequences with indication of T582 and region (646–716). (**B**) Alignment of IR, IGF-IR and IRR sequences with indication of L868 and R869. (**C**) Domain models of the 6xHis-tagged T582-TK(N), LR-TK(N), and AED-TK(N) alanine mutants or chimera with partial tyrosine kinase domain swapping TK(N). (**D**) Quantitative analysis of the activation of indicated constructs by alkali in comparison with IRR-TK(N) as 100%. The Western blots with samples from in vitro autophosphorylation assays were blotted with anti-pIR/IRR antibodies and, after stripping, with anti-IR/IRR antibodies. Quantitative analysis of the activation of the constructs performed as described in Materials and Methods. Asterisks indicate *p* < 0.05 in comparison with the IRR-TK(N). Values are means ± SE (*n* ≥ 4).

To analyze the role of fibronectin type-III repeats regions (FnIII-1, FnIII-2, and FnIII-3) in detail, we have constructed plasmids with alanine mutagenesis of potentially important amino acid residues, which were identified by the multiple alignment of the fibronectin domains of IR, IGF-IR, and IRR sequences from different species (Figure 1A,B). We searched for the residues that were evolutionarily conserved in IRR but differed significantly from those of IR or IGF-IR by polarity, size, or charge. Three amino acid residues T582, L868, and R869 in the FnIII-1 and FnIII-3 domains of IRR were thus identified and mutated to alanine (Figure 1A,B). We made two mutant constructs: one T582-TK(N) with single mutation in T582 and another LR-TK(N) with double mutations in L868 and R869 (Figure 1C).

The pH-sensing activity of the mutants was analyzed utilizing the previously developed in vitro autophosphorylation assay that allows quantitative assessment of tyrosine kinase activation [14,21]. To facilitate this assay, all of the constructs were made on the basis of the IRR-TK(N) chimera (Figure 1C), which contained the N-terminal fragment of the catalytic tyrosine kinase domain of IR (from R1027 to M1103) swapped with those of IRR [14].

The activity of T582 mutant decreased to 64 ± 2% as compared to the wild-type IRR (Figure 1D), whereas the double mutation of both L868 and R869 did not produce any effect on the pH sensing activity (Figure 1D).

According to the published crystal structure of the IR ectodomain that represents a Λ-shape head-to-tail symmetrical dimer, L1 and C domains of one monomer come in contact with FnIII-2 and FnIII-3 domains of the other, while its L2 domain is close to the FnIII-1 domain of the opposing subunit [23]. The structure of a small region (663–734) at the C terminus of alpha IR subunit within FnIII-2 domain was not completely resolved by crystallography, presumably due to its disordered nature [24]. Identities of this region between IRR and IR is about 39% (Figure 1A). We tested a potential significance of this disordered region by analyzing the construct named AED_TK(N) where the region (646–716) of IRR by partial swapping this region by identical part from IR (Figure 1C), and this chimera showed huge decreased activity to 19 ± 4% (Figure 1D).

**Figure 2:**
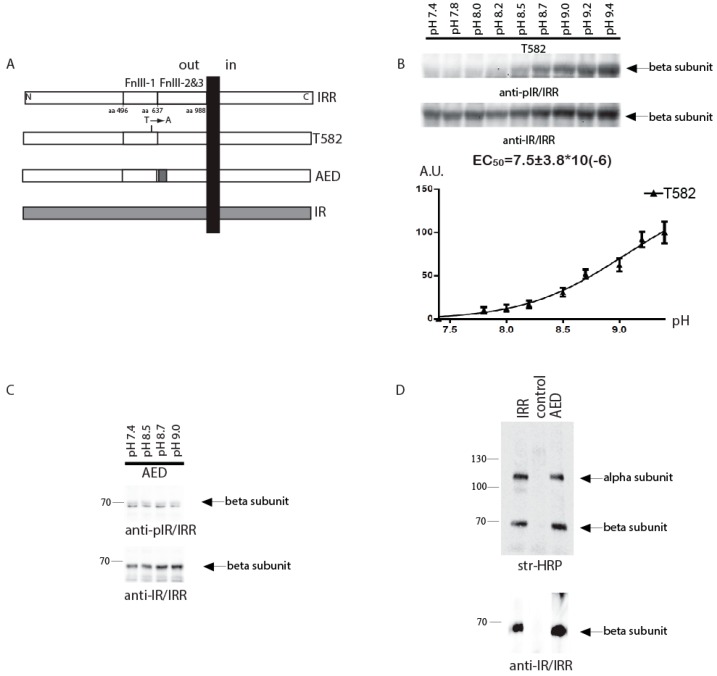
The pH dependence of IRR chimeric mutants. (**A**) Domain models of the HA-tagged T582 and AED mutants and chimeras. (**B**) HEK 293 cells were transfected with indicated plasmid and incubated for 10 min with PBS medium adjusted to the indicated pH by 60 mM Tris-HCl buffer, followed by lysis and blotting with anti-pIR/IRR and anti-IR/IRR antibodies after stripping. Western blot phosphorylation signals were quantified and normalized according to the anti-IR/IRR signals. On each graph normalized signals were plotted versus pH of the tested solutions. On each plot, the Y-axis shows percentage of the maximum average value of activation at pH 9.4. The values of EC_50_ were calculated by GraphPad software. Values are means ± SE (*n* = 4). (**C**) The pH dependence of AED chimera phosphorylation. Pictures represented at least four independent experiments. (**D**) Cell surface expression of AED chimera as described in Materials and Methods.

We further tested the effect of T582 and AED mutations on the pH dependence of IRR activation in the intact cell assay (Figure 2A) with varied pH of the conditioned media from 7.4 to 9.4. In this assay, the constructs contained the unchanged intracellular tyrosine kinase domains of IRR. We estimated the Hill’s slope (H) and point of half effect (EC_50_) for tested constructs and compared them with the characteristics of the wild type IRR that equal EC_50_ = 4.3 ± 0.5 µM and H = 2.3 ± 0.3 [14]. The Hill’s slope for the T582-IRR mutant response was 1.1 ± 0.2 that indicated a loss of positive cooperativity. To estimate the half-effect for T582-IRR, we used nonlinear regression of “one site-specific binding” type and obtained EC_50_ = 10.7 ± 2.6 µM for this mutant, which was shifted towards alkalinity, as compared to wild type IRR (Figure 2B).

For the AED-IRR construct, we failed to find any significant response to alkali treatment (Figure 2C), although we detected a low level of basal phosphorylation of this construct. We checked the cell surface expression of AED-IRR chimera and found that it is comparable to that of wild type IRR (Figure 2D). Similar data were obtained previously for the chimera with all three fibronectins repeat domains being swapped with those of IRR. They showed no activity in the intact cell assay, but small and detectable activity in the in vitro autophosphorylation assay [21]. Together, these findings indicate that either the in vitro autophosphorylation assay is more sensitive than the intact cell assay, or the IR catalytic domain is more active than the IRR one.

**Figure 3:**
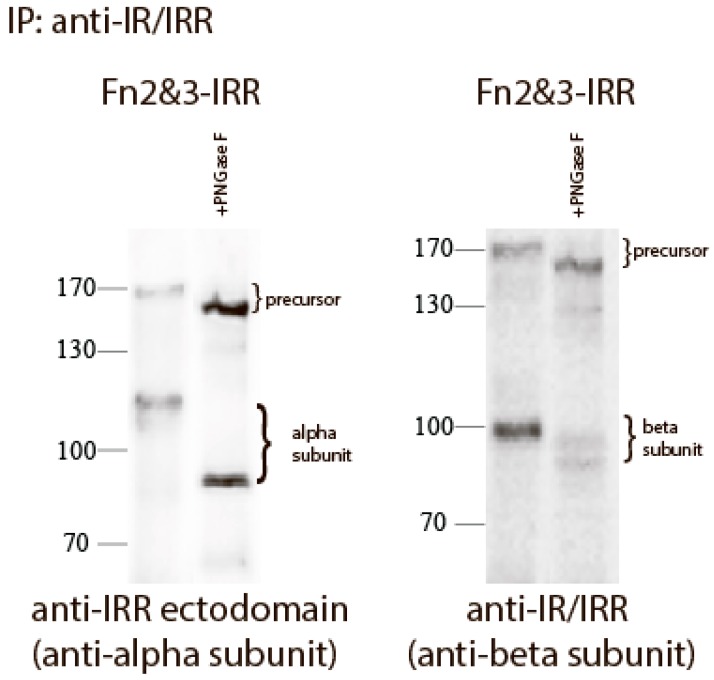
Treatment of Fn2&3-IRR with PNGase. Proteins from HEK 293 transfected cells was immunoprecipitated with anti-IR/IRR antibodies. The immunoprecipitates were treated without or with PNGase. After digestion, the reaction mixture was analyzed by Western blotting with indicated antibodies.

We reported previously that the replacement of both FnIII-2 and FnIII-3 domains of IRR with those of IR (Fn2&3-IRR construct) completely abolished its pH sensing and also shifted the size of its beta-subunit from about 70 to about 100 kDa [21]. This effect can be explained by N-linked and O-linked glycosylation of FnIII-2 and FnIII-3 domains of IR [8,25], whereas IRR is less glycosylated [25]. To estimate the N-linked oligosaccharide content of the chimera Fn2&3-IRR, we treated the mutant protein expressed in HEK 293 cells with Peptide-N-Glycosidase F (PNGase F) that catalyzes the cleavage of N-linked oligosaccharides between the innermost GlcNAc and asparagine residues of high mannose, hybrid, and complex oligosaccharides from N-linked glycoproteins. After digestion, the molecular mass of Fn2&3-IRR chimera decreased from about 120 to 80 kDa for alpha subunit, and from about 100 to 90 kDa for beta subunit (Figure 3).

**Figure 4:**
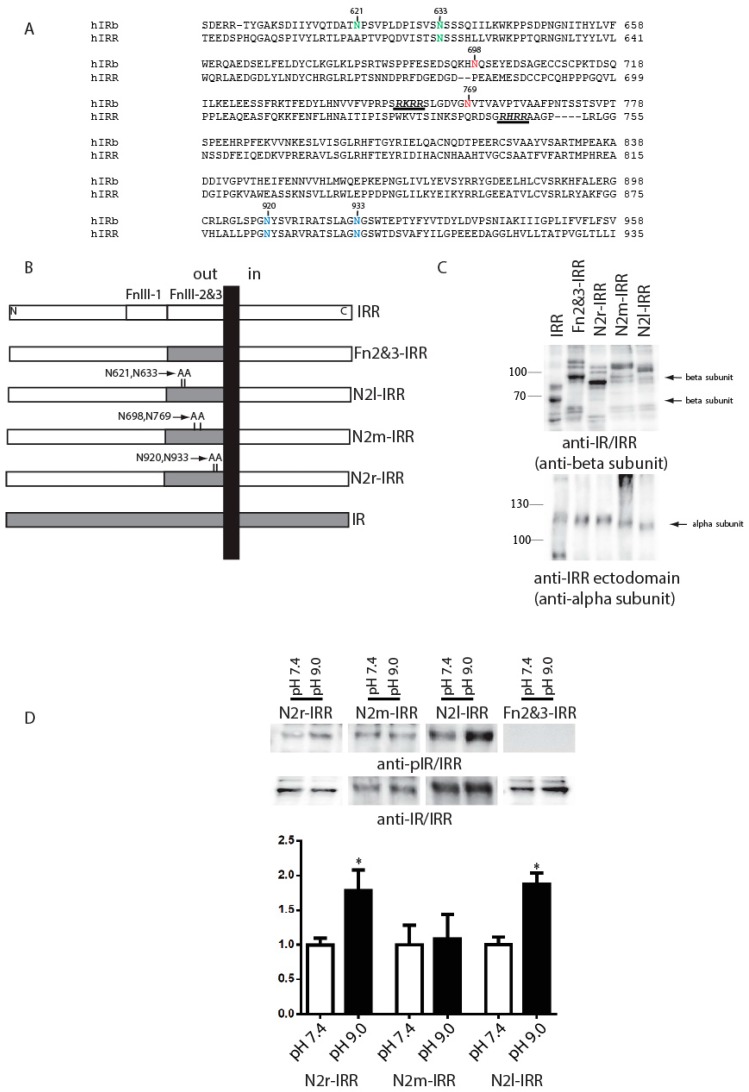
The role of glycosylation within fibronectin repeats domains. (**A**) Alignment of human IR isoform b and human IRR sequences with indication of mutated resides. N2l (left), N2m (middle) and N2r (right) mutations indicated by green, red, or blue colors, correspondently. Sites of proteolysis also indicated by underlying; (**B**) Domain models of the HA-tagged Fn2&3-IRR, N2l-IRR, N2m-IRR, and N2r-IRR alanine mutants or chimeras; (**C**) Western blotting of indicated constructs with anti-IR/IRR antibody (against C-end of IRR) and with anti-IRR ectodomain antibody (against alpha-subunit of IRR); (**D**) Quantitative analysis of the activation of indicated constructs by alkali. For each constructs basal phosphorylation at pH 7.4 indicated as 100%. Transfected cells were incubated with two set of Tris-buffered physiological saline solutions with pH from 7.4 or 9.0. Lysates of transfected cells were directly analyzed by Western blotting with anti-pIR/IRR antibodies and after stripping with anti-IR/IRR antibodies. For the quantitative analysis of Western blots we used Fusion Solo system (Vilber Lourmat, France). Asterisks indicate *p* < 0.05 in comparison with the basal phosphorylation at pH 7.4 of same constructs. Values are means ± SE (*n* ≥ 4).

The significant difference in the extent of IR and IRR glycosylation suggested that the carbohydrate moiety of IRR may play a role in its alkali-dependent activation. To test this possibility, we removed three potential sites for N-glycosylation in Fn2&3-IRR chimera. Three different constructs with double mutations of two different asparagines in FnIII-2 and FnIII-3 domains to alanines were named N2r-IRR, N2m-IRR and N2l-IRR (Figure 4A,B). The corresponding residues in IR are N-glycosylated [26]. Western blotting of cells, transfected with N2r-IRR, N2m-IRR, N2l-IRR and Fn2&3-IRR constructs, with anti-beta subunit (anti-IR/IRR) and anti-IRR alpha subunit antibodies revealed a shift of the bands of alpha-subunit in N2l-IRR and beta-subunit in N2r-IRR constructs (Figure 4C). These mutated constructs were tested for alkali-dependent activation in the intact cell assay. We found that the N2r-IRR and N2l-IRR mutants partially restored their alkali-sensing property while N2m-IRR and Fn2&3-IRR did not show any activity (Figure 4D).

## 3. Discussion

Alkali-sensing receptor tyrosine kinase IRR is a close relative of the insulin receptor. They have identical domain structures and high protein sequence homology. Yet, they react to totally different agents, a peptide and hydroxyl-anion without any cross-reactivity. In an attempt to identify the key structural differences between these receptors that define the IRR alkali-sensing properties, we analyzed the impact of single point mutations, as well IRR/IR domain swapping on their activity. We also addressed the question of whether the carbohydrate moiety of these receptors may contribute to their interaction with agonist. The interpretation of our results is based on the assumption that the IRR structure resembles the published three-dimensional structure of the IR ectodomain [23], which is a lambda shaped symmetrical head-to-tail complex of two disulphide-linked monomers.

Our previous data suggested that multiple domains of the IRR extracellular N-terminal region contribute to the receptor alkali-sensing property, the L1C and three FnIII domains being the most important. The activation of IRR has a positive cooperativity with Hill’s coefficient of about 2.3 [21]. We proposed that IRR contain two pH sensing sites, the first site being located between L1 and C domains that contact FnIII-2 and FnIII-3 domains of the adjacent monomer, and the second site lying between L2 domain and FnIII-1 domain of the opposing monomer [21]. The results of this study expand our knowledge about the pH-sensing sites of IRR.

**Figure 5:**
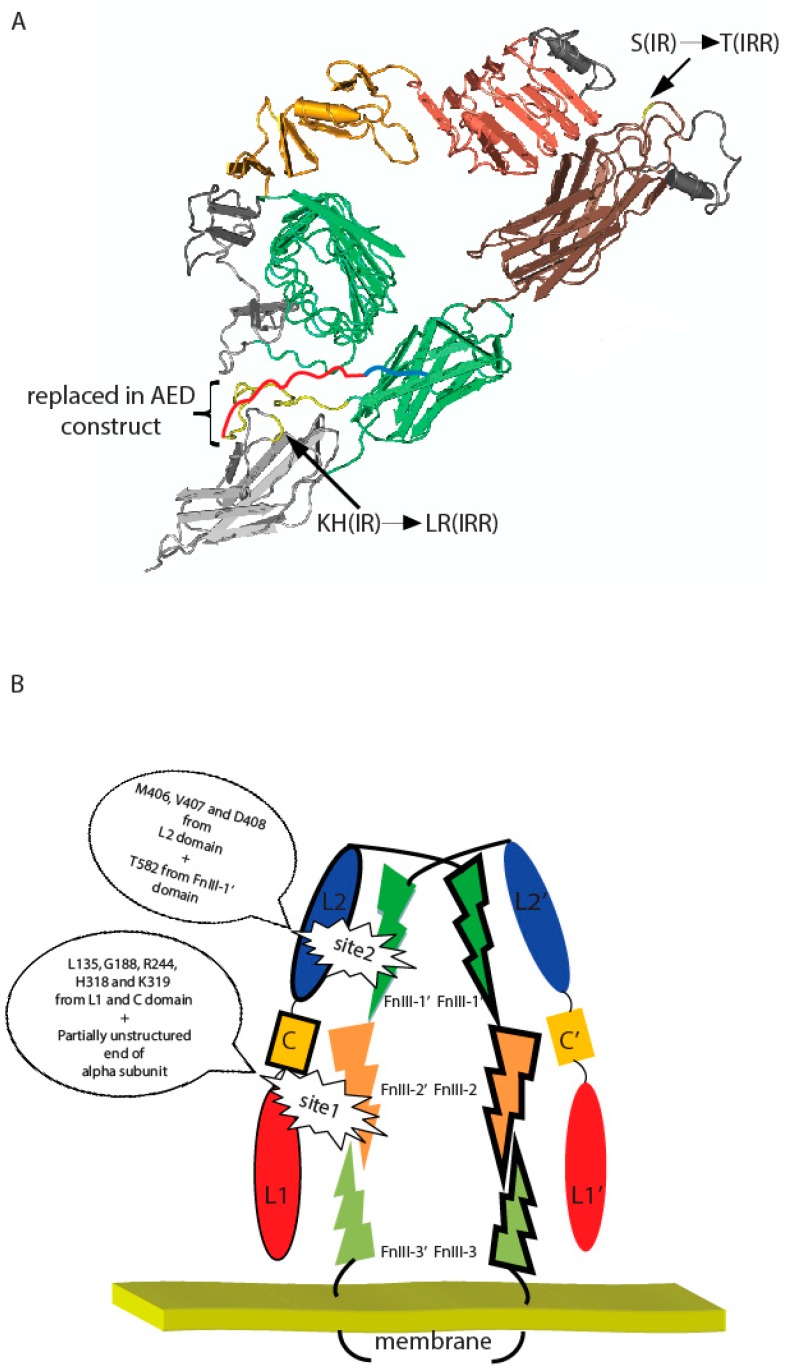
(**A**) Positions of indicated T582, L868, R869, and region (646–716) within fibronectin repeats domains and their IR substitutes on a putative three-dimensional model of IRR built according to the described structure of IR ectodomain [23] with Cn3D 4.3.1 software (NCBI, Bethesda, MD, USA). Region, which were swapped in AED chimera construct, was indicated by yellow color (presented in IR ectodomain structure) and red color (unstructured part of IR alpha subunit). Blue color showed rest of unstructured part of IR alpha subunit. (**B**) Two-site activation model of IRR. Important residues are indicated. Domains indicated by different colors. Monomers indicated by trait.

Mutation of the T582 residue of the FnIII-1 domain significantly reduced the IRR activity and was also accompanied by a loss of positive cooperativity. This residue is located closely to the top of the dimer “arch” and can physically face the L2 domain of the second subunit (Figure 5A). We earlier showed that mutation of the residues M406, V407, and D408 in the L2 domain significantly reduced IRR pH-sensing activity and also abolished its positive cooperativity [21]. We may therefore assume that M406, V407, D408, and T582 residues are key components of the second center of pH sensing in IRR (Figure 5B).

When the “unstructured” region within FnIII-2 domain of IRR (residues 646–716) was swapped with the corresponding fragment of IR (663–734), a large negative effect on alkali sensing was observed. Homological region in IR ectodomain structure is partially unstructured, which indicated great mobility of this part (Figure 5A). We suggest this region in IRR functions as an “internal” ligand by interacting with adjacent L1 and C domain of the second subunit upon alkali treatment and, together with previously mapped amino residues L135, G188, R244, H318, and K319 of L1 and C domains, forms the first and primary site of pH sensing in IRR (Figure 5B).

Our data suggest that not only the polypeptide backbone of IRR, but also its carbohydrate coat is important for pH sensing. It is known that IRR is significantly less glycosylated than IR or IGF-IR, mostly in FnIII-2 and FnIII-3 domains [25,27]. Our current experiments with N2r-IRR, N2m-IRR, N2l-IRR, and Fn2&3-IRR constructs shed some light on possible reasons for this phenomenon and indicated that additional glycosylation in alpha- and beta-subunits of IRR produces a strong negative effect on its pH sensing. Perhaps, a thicker carbohydrate coat provides a steric hindrance to rapprochement of IRR fragments during the major conformational change that accompany IRR activation. We suggest that additional glycosylation of IR and IGF-IR can completely abolish potential pH-sensing of these receptors.

Altogether, our previous and current data suggest complex multipoint interactions inside IRR ectodomain under alkali-induced conformational changes within the IRR ectodomain. Although we identified certain parts and residues needed for pH-sensing, the precise molecular mechanism of IRR activation would require future detailed studies of the receptor structure by modern physical methods.

## 4. Materials and Methods

### 4.1. IRR/IR Chimeric Receptors and Mutagenesis

The chimeras of human IRR and IR with partial ectodomain or tyrosine kinase domain swapping were obtained by cloning using the PCR strategy, as described in [10]. To introduce point mutations, we used the megaprimer PCR approach with mutated oligonucleotides [14]. The structure of all the constructs was confirmed by DNA sequencing (Supplement Materials). All of the constructs were based on pcDNA 3.1 neo vector (Invitrogen, Thermo Fisher Scientific, Waltham, MA, USA).

### 4.2. Cell Cultures, Transfections and Tyrosine Phosphorylation Analysis in Intact Cells

HEK 293 cells were cultured in DMEM supplemented with 10% fetal bovine serum (Hyclone, GE Healthcare, Chicago, IL, USA), 1% penicillin/streptomycin, and 2 mM l-glutamine. The cells with confluent density of about 50% were transfected by unifectin-56 (Unifect Group, Moscow, Russia), according to manufacturer’s protocol. In 36–40 h after transfection, cells were washed with serum-free F-12 and incubated for 3 h in serum-free F-12 containing 1% penicillin/streptomycin in a CO_2_ incubator. The cells were further incubated in PBS with 60 mM Tris-HCl with different pH values at room temperature and lyzed in the SDS-PAGE sample buffer (75 mM Tris-HCl pH 6.8, 1.5% SDS, 150 mM b-MeEtOH and 15% Glycerol).

To analyze the pH dependence of the mutants activation, transfected cells were incubated with a set of Tris-buffered physiological saline solutions with pH varied in the range from 7.4 to 9.4 in small increments. The transfected cells were lyzed with the sample buffer, separated by electrophoresis, and analyzed by Western blotting with anti-pIR/IRR antibodies [14]. The blots were further stripped and stained again with anti-IR/IRR C-terminal antibody. The blots were visualized by chemiluminescence that was captured with Fusion Solo system.

The ratio of integral density of the phosphorylated receptor (pIR/IRR signal) to the total receptor (IR/IRR antibody signal) was plotted versus pH. The effect is shown as a percentile of the strongest signal at pH 9.4 for each mutant. EC_50_ and a Hill slope were calculated by GraphPad 6.0.1 software (GraphPad Software, La Jolla, CA, USA) that analyzed the data through a nonlinear regression of “one site-specific binding with Hill slope”.

### 4.3. Autophosphorylation Assay In Vitro

HEK 293 cells were transfected with plasmids encoding IRR or mutant chimeras with C-terminal 6xHis-tag, essentially as described [14]. Two days after the transfection, the cells were washed by serum-free F-12 and further incubated for 3 h in serum-free F-12 containing 1% penicillin/streptomycin. The cells were then lyzed in ice-cold lysis buffer (50 mM Hepes-KOH pH 7.4, 150 mM NaCl, 1% Triton X-100, 1 mM PMSF). Cell extracts were centrifuged at 15,000× *g* for 15 min and the supernatants were further incubated with Ni-NTA agarose (Qiagen, Venlo, Netherlands) at +4 °C for 90 min. The matrices were further washed three times with the lysis buffer and were incubated with a cold elution buffer (100 mM Hepes-KOH pH 7.4, 150 mM NaCl, 100 mM imidazole, 15 mM MgCl_2_, 0.1% Triton X-100 and 1 mM Na_3_VO_4_) at +4 °C for 90 min. Two samples of the eluates (80 µL) were supplemented with 20 µL of 1 M Tris-HCl pH 7.1 or 9.1 (at +25 °C) and incubated for 30 min on ice. ATP solution was further added to the final concentration of 100 nM and the samples were placed at +25 °C for 5 min. To stop the autophosphorylation reaction, 5× SDS-loading buffer was added and the samples were boiled. Then, eluates were blotted with anti-pIR/IRR antibodies. To normalize the data for the actual receptor protein amount, the blotting membranes, after staining with anti-pIR/IRR antibodies, were stripped with stripping solution (50 mM Tris-HCl, pH 6.8, 2% (*w/v*) SDS, 100 mM 2-mercaptoethanol) and reprobed with anti-IR/IRR antibodies. The differences of normalized phosphosignals after alkaline and neutral pH-treatment for chimeras or mutants were compared with the same data for IRR-TK(N) construct, which was used as the 100% reference.

### 4.4. Antibodies and Western Blotting

Rabbit anti-IR/IRR antibodies were raised against the human IRR C-terminal cytoplasmic domain (aminoacid residues 961-1297) expressed in bacteria as GST-fusion protein. The anti-pIR/IRR antibodies were raised against KLH-coupled peptide CGMTRDVpYETDpYpYRKGGKGL from the activation loop of IRR, as described in [10]. The anti-IRR ectodomain antibodies were raised against the mouse IRR ectodomain (aminoacid residues 539–686) expressed in bacteria as GST-fusion protein. The lysates and eluates were separated by electrophoresis in 8% SDS-PAGE followed by blotting onto ECL-grade nitrocellulose (Amersham, GE Healthcare, Chicago, IL, USA) as described in [28]. The bound antibodies were detected with anti-rabbit HRP-conjugated secondary antibodies (Jackson ImmunoResearch Laboratories, West Grove, PA, USA following visualization with SuperSignal Chemiluminescent Substrate System (Pierce, New Brighton, MN, USA). For the quantitative analysis of Western blots we used Fusion Solo system (Vilber Lourmat, France). The captured images were manually selected in rectangles and further analyzed by densitometry with Fusion software (Vilber Lourmat, France) or ImageJ program (National Institutes of Health (NIH), Bethesda, MD, USA) the background being subtracted by selecting non-stained blot areas. Final calculations were made using GraphPad 6.0.1 software (GraphPad Software, La Jolla, CA, USA).

### 4.5. Cell Surface Expression of IRR/IR Mutants and Chimeras

HEK 293 cells were transfected with the AED chimera and IRR construct. After starvation, the cells were biotinylated with Biotin 3-sulfo-*N*-hydroxysuccinimide ester sodium salt (Sigma, USA) according to protocol provided by Pierce Company (New Brighton, MN, USA) (http://www.piercenet.com/files/0237dh4.pdf), then lysed, and immunoprecipitated with anti-IR/IRR antibody. The precipitates were stained with anti-IR/IRR antibody or streptavidin-HRP (Amersham, GE Healthcare, Chicago, IL, USA).

### 4.6. Deglycosylation of Fn2&3-IRR Chimera

HEK 293 cells were transfected with plasmids encoding Fn2&3-IRR chimera. Two days after the transfection, the cells were washed by serum-free F-12 and cells were further lyzed in the ice-cold lysis buffer (50 mM Hepes-KOH pH 7.4, 150 mM NaCl, 1% Triton X-100, 1 mM PMSF). Cell extracts were centrifuged at 15,000× *g* for 15 min and the mutant protein was precipitated by incubation with rabbit anti-IR/IRR antibodies at +4 °C for overnight, followed by protein A agarose incubation at +4 °C for 90 min. The matrices were washed three times with the lysis buffer and the absorbed protein was eluted with 1% SDS solution. Then, half of eluate was incubated with PNGase F (New England Biolabs) according manufacture’s instruction for deglycosylation in denaturing conditions. Samples with or without PNGase F treatment were analyzed by Western blotting with anti-IRR ectodomain antibody and anti-IR/IRR antibody.

## Supplementary Materials

Supplementary materials can be found at http://www.mdpi.com/1422-0067/18/11/2461/s1.
